# Yeast and Fertility: Effects of *In Vitro* Activity of *Candida* spp. on Sperm Quality

**Published:** 2018

**Authors:** Elizabeth Ximena Castrillón-Duque, Jennifer Puerta Suárez, Walter Dario Cardona Maya

**Affiliations:** - Grupo Reproducción, Departamento de Microbiología y Parasitología, Facultad de Medicina, Universidad de Antioquia, Medellín, Antioquia, Colombia

**Keywords:** *Candida albicans*, *Candida glabrata*, Male infertility, Semen, Spermatozoa

## Abstract

**Background:**

*Candida* spp. causes semen candidiasis, the most important sexually transmitted fungal infection; this microorganism affects male fertility potential and could alter oocyte fertilization. The *in vitro* effects of the yeasts *Candida albicans* and *Candida glabrata* and their soluble factors of fungal metabolism on semen quality were studied.

**Methods:**

*Candida* strains (2, 0.5 and 0.05 McF) and their soluble factors were incubated for 3 *hr* with selected spermatozoa. Conventional (Viability and motility) and functional parameters (Mitochondrial membrane potential, membrane integrity, detection of reactive oxygen species and DNA fragmentation) were quantified in 35 semen samples. In addition, human spermatozoa were incubated under capacitating conditions with *Candida* spp. and soluble factors. Finally, spermatozoa were incubated with mannose before incubation with either yeast to block sperm and yeast interaction. Data was analyzed using Friedman test, and p<0.05 was considered significant.

**Results:**

The conventional sperm parameters were statistically affected by the two yeast strains after 3 *hr* and their effect was maintained until the 24 *hr* incubation. However, the functional parameters were altered, this change was not statistically significant. Pretreatment of spermatozoa with mannose decreased the effect of *Candida* spp.

**Conclusion:**

The presence of *C. albicans* or *C. glabrata* affects seminal parameters. The effect is related to incubation time and yeast concentration, it can be supposed that the yeast sperm interaction is mediated through the mannose sperm receptor.

## Introduction

Male fertility potential is related to the quality of the sperm present in the ejaculate. During ejaculation, semen is produced from a concentrated suspension of spermatozoa (∼10%) stored in the paired epididymides, vas deferens and fluid secretions from the accessory sex organs (1). Seminal fluid can hold and transport bacteria, fungi, parasites and viruses that provide adequate conditions for microorganism survival in seminal plasma (2–5).

The male urogenital tract is colonized by these etiological agents that sometimes interference with sperm functions and cause infertility (6). The effect of viral and bacterial infections is commonly evaluated, while those caused by fungi are undervalued (7, 8). *Candida* spp., *Aspergillus* spp., *Cryptococcus* spp., and *Coccidioides* spp. account for more than 90% of the systemic mycoses reported and are caused by fungi. In the male reproductive tract, fungus causes urethritis, balanoposthitis, ulcers, pseudotumor, orchitis and prostatitis (7, 9, 10). Candidiasis is considered as the only sexually transmitted yeast infection; it is caused by *Candida* spp., *Candida albicans* and *C. glabrata* as the most prevalent species (11), and this infection is much more common in women than in men (2, 11). However, men can act as infection reservoir. Contact of *C. albicans*, or the soluble factor of fungal metabolism with seminal plasma alters sperm quality, either in the male reproductive tract (before ejaculation) or in the female reproductive tract (after ejaculation or sperm capacitation) (8, 12, 13).

Some *in vitro* studies report decreased motility and sperm viability, increased sperm DNA fragmentation, and alteration of the mitochondrial membrane affecting the potential of male fertility and nonspecific agglutinations (8, 12, 13). Conversely, the effects of *C. glabrata* on sperm cells are still unknown.

Burrello et al. (8, 14) reported that failure in the assisted reproductive program could occur in patients with *C. albicans* infections due to the increase of DNA fragmentation and apoptosis, but without affecting conventional sperm parameters. Even gradient methodologies commonly used in in semen processing reproductive programs cannot remove *C. albicans* contamination (8, 14). In addition, Tian et al. (13) demonstrated that *C. albicans* infection affects sperm motility and damages the ultrastructure of sperm cells.

Based on the previous demonstrations, within the diagnosis of yeast infection, it is mandatory to: i) determine the effect of *C. albicans* and *C. glabrata* on functional sperm parameters; ii) determine the possible receptor involved in the sperm and *Candida* spp. interaction, since this is important for medical and reproductive programs. These results will demonstrate further knowledge about *Candida* spp. infections and appropriate treatments to prevent possible negative results in the procedure of assisted reproduction.

The aim of this study was to evaluate the *in vitro* effects of *C. albicans*, *C. glabrata* and their soluble factors of fungal metabolism on sperm quality.

## Methods

### Yeast:

*C. albicans* and *C. glabrata* strains (kindly donated by the Institución Universitaria Colegio Mayor de Antioquia. Medellín, Colombia) were conserved on Sabouraud agar (Thermo Fisher Scientific, Waltham, MA, USA) at 4°*C*. Two days before use, each strain was reactivated in brain heart infusion (BHI, Thermo Fisher Scientific, Eugene, OR, USA) at 37°*C*.

### Sperm samples:

Thirty-five semen samples were donated by apparently healthy volunteers, aged between 18 and 32 years, collected by masturbation after a sexual abstinence period of 3 to 5 days and analyzed during the first hour after sample collection at the Reproduction Group, Medical School of the University of Antioquia. The conventional seminal parameters were determined in each semen sample following the guidelines established in the semen processing manual of the World Health Organization (WHO), and sperm concentrations were determined using a Makler chamber (Sefi-Medical Instruments). All the samples had seminal parameters equal to or above the lower reference limits established by WHO in 2010. Exclusion criteria for study participation were any history of urogenital surgery and leuko-cytospermia as well as self-reported illnesses or use of medication in the three months immediately preceding the study.

### Sperm selection:

Motile sperm were selected using the gradient technique (PureSperm® Nidacon, Gothenburg, Sweden). Briefly, in a 15 *ml* conical tube, 500 *μl* of the denser phase (PureSperm^®^ 80), 500 *μl* of the less dense phase (PureSperm^®^ 40) and 1000 *μl* of semen over both phases were added, and the mixture was centrifuged 20 *min* at 300 *g*. Finally, the supernatant was removed and the motile sperm was re-suspended in 1 *ml* Ham’s-F10 medium (Gibco Life Technologies, Breda, Netherlands).

### Yeast inoculum and soluble factor of fungal metabolism:

2, 0.5 (1–5×10^6^ *yeast/ml*) and 0.05 Mc Farland (McF) yeast inoculums in phosphate-buffered saline (PBS, Gibco Life Technologies, Grand Island, NY, USA) were incubated with three different relation between motile sperm and *Candida* spp. *Candida* and sperm ratios were 1 to 1 (0.5 McF), 1 to 10 (0.05 McF) and 4 to 1 (2 McF). The 0.05 McF inoculum was prepared from a 1:10 dilution of 0.5 McF.

Soluble factors of fungal metabolism (SFFM) of *C. albicans* and *C. glabrata* were obtained after a 0.5 McF PBS (Gibco Life Technologies) incubation for 1 *hr* at 37°*C*. Next, the medium was centrifuged for 5 *min* at 2300 *g* and subsequently filtered through a 0.2 *μm* membrane (Advantec Industries, Yinzhou District, Ningbo, China). The filtrate was aliquoted and stored at −20°*C* until used.

### Evaluation of the effect of C. albicans, C. glabrata and SFFM on conventional sperm parameters:

Three concentrations (2, 0.5 and 0.05 McF) of each yeast strain and 0,5 McF SFFM were incubated with five million selected sperm and the effect of incubation over conventional sperm parameters was determined after 0, 1, 2 and 3 *hr* of incubation at 37°*C* following intervals previously described (8, 13). The results are expressed as the percentage of change in viability and progressive sperm motility relative to the control (Spermatozoa incubated in PBS).

### Evaluation of the effect of C. albicans, C. glabrata and SFFM on capacitated spermatozoa:

Five million selected sperm were incubated in four different conditions: i) Capacitation medium (MC, Ham's F10-Gibco Life Technologies-with 35 *mg/ml* bovine serum albumin (Sigma-Aldrich, St. Louis, MO, USA)), ii) Non-capacitation medium (Ham's F10-NC), iii) Capacitation medium with 0.5 McF SFFM (MC-FS), and iv) Capacitation medium with *Candida* spp., 0.05 McF (MC-0.05 McF). Finally, the progressive motility in the initial time (0 *hr*), and 6, 18 and 24 *hr* post capacitation was evaluated.

### Evaluation of the effect of the SFFM of C. albicans and C. glabrata on the functional parameters:

Fifteen million previously selected sperm were incubated with 0.5 McF SFFM of *Candida* spp. in a ratio of 1:1 *v/v*, for 3 *hr* at 37°*C*. The control was 15 million spermatozoa incubated with PBS. The following functional tests were evaluated, analyzing a total of 10000 events for each sample according to previously established protocols (15–22).

### Mitochondrial membrane potential (ΔΨ_m_):

One million sperm were incubated with 3,3′-dihexyloxacarbocyanine iodide DIOC_6_ (final concentration 10 *nM*, Molecular Probes Inc., Thermo Fisher Scientific) and propidium iodide (PI, final concentration 12 *μM*, Molecular Probes Inc., Thermo Fisher Scientific) in the dark (30 *min*, 25°*C*). The samples were washed in PBS, centrifuged at 180 *g* for 5 *min*, and the pellets re-suspended in PBS and subjected to flow cytometry. Data were acquired as the percentage of living spermatozoa showing high (ΔΨ_m_^high^) or low (ΔΨ_m_^low^) green fluorescence, and dead spermatozoa-red fluorescence (19, 21).

### Evaluation of plasma membrane integrity:

One million sperm were incubated in Sybr-14 and PI (Final concentrations of 1 *μM* and 12 *μM*, respectively, The LIVE/DEAD^®^ Sperm Viability Kit, Molecular Probes Inc., Thermo Fisher Scientific) in the dark (30 *min*, 25°*C*), washed once and resuspended in PBS prior to flow cytometry analysis. The data are expressed as the percentage of viable spermatozoa (19, 21).

### Lipid peroxidation assay:

One million sperm were incubated in the dark (30 *min*, 25°*C*) with BODIPY C11 (Final concentration 6.6 *μM*, Molecular Probes Inc., Thermo Fisher Scientific), washed and re-suspended in PBS before flow cytometry analysis. The results are expressed as the percentage of spermatozoa exhibiting a green fluorescence response (15).

### Production of intracellular reactive oxygen species:

One million sperm were incubated with PI (final concentration 12 *μM*) and 2′,7′-dichlorodihydro-fluorescein diacetate (DCFH-DA, Sigma-Aldrich, St Louis, MO, USA, final concentration 1 *μM*) in the dark for 5 *min*, at 25*ºC*, washed three times with PBS (180 *g*, 5 *min*) and the pellets resuspended in PBS before being analyzed by flow cytometry. The results are expressed as the percentage of spermatozoa exhibiting the green fluorescence response (23–25).

### Sperm chromatin structure assay:

Five million sperm were suspended in 200 *μl* of TNE buffer (Tris-HCl, NaCl and EDTA, pH=7.4). Before reading on the cytometer, 200 *ml* of acid detergent solution (HCl, NaCl, Triton X-100, water, pH=1.2) was added and 30 *s* after, the sperm were stained with 600 *μl* of acridine orange (Sigma-Aldrich) staining solution (6 *μg/ml*). The ratio of single stranded DNA (red) to single plus double stranded DNA (green) media fluorescence intensity (MFI) is expressed as the DNA fragmentation index (DFI) (19, 21).

### Detection of membrane phosphatidylserine exposure:

An aliquot of 2×10^6^ spermatozoa was re-suspended in binding buffer, labeled with Annexin-FITC plus PI, incubated for 15 min in the dark, followed by the addition of binding buffer (10x: 0.1 *M* Hepes pH=7.4, 1.4 *M* NaCl; 25 *mm* CaCl_2_) and then immediately transferred to a flow cytometer and analyzed according to the manufacturer’s instructions (eBioscience Apoptosis Detection Kit FITC; BD, Thermo Fisher Scientific). The results are expressed as percentage of cells for each population of interest, phosphatidylserine exposure and PI positive (23).

### Blockage of the mannose receptor:

Sperm cells were incubated with mannose (Sigma-Aldrich, St. Louis, MO, USA, 100 *μM*) for 1 *hr* and subsequently, the sperm cells were incubated with each yeast (0.5 McF). The results are expressed as the percentage change in progressive sperm motility relative to the control (spermatozoa incubated in PBS) at 0 and 3 *hr*.

### Statistical analysis:

The flow cytometry results of each functional parameter were processed using the FlowJo program 7.6 (TreeStar, San Carlos, CA, USA). Descriptive statistics were applied (Median and range). For the analysis of the conventional and functional parameters, a non-parametric Friedman was performed (Friedman test) considering a statistical significance of p<0.05. The assumption of normality was checked by Kolmogorov-Smirnov, Shapiro-Wilk test and D’Agostino-Pearson using Prism 5.0 (GraphPad Software, San Diego, CA) statistical software.

## Results

**Figure 1.**
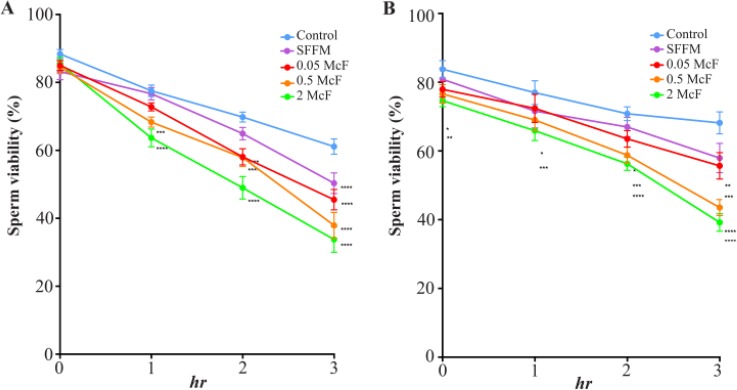
Effect of *Candida* spp. on sperm viability. *C. albicans* (a), *C. glabrata* (b) at three concentrations (0.05, 0.5 and 2.0 McF) and their SFFM on sperm viability at 0, 1, 2 and 3 *hr* of incubation. The data are presented as median and a range of seven (*C. albicans*) or three (*C. glabrata*) independent experiments with duplicate determinations. *: p<0,05; **: p<0,01; ***: p<0,001, ****: p<0,0001. SFFM: Soluble factors of fungal metabolism

The effect of *C. albicans*, *C. glabrata* strains and their 0.5 McF SFFM on sperm viability is shown in figure 1. *C. albicans* progressively decreased sperm viability to 45% (2 McF, p<0.0001) and their SFFM to 18% (p< 0.0001) based on the control (Figure 1A), whereas *C. glabrata* strain achieved a maximum reduction of 42% (2 McF, p<0.0001) and their SFFM of 15% (p<0.01) with respect to the control (Figure 1B).

**Figure 2.**
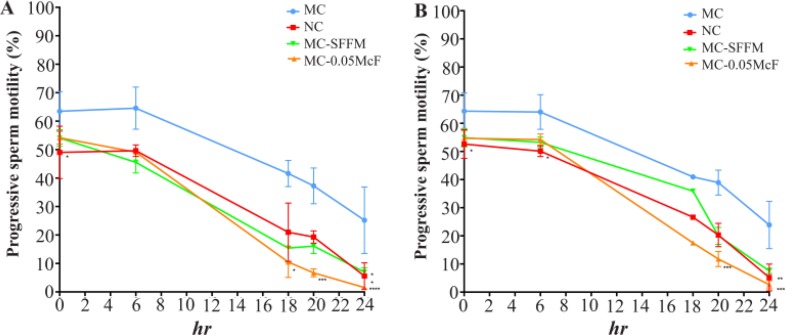
Effect of *Candida* spp. on sperm motility. *C. albicans* (a) and *C. glabrata* (b) at three different concentrations (0.05, 0.5 and 2.0 McF) and their SFFM on the motility of progressive sperm at 0, 1, 2 and 3 *hr* of incubation. The data are presented as median and a range of seven (*C. albicans*) or three (*C. glabrata*) independent experiments with duplicate determinations. *: p<0,05; **: p<0,01; ***: p< 0,001, ****: p<0,0001. SFFM: Soluble factors of fungal metabolism

The effect of the strains of *C. albicans*, *C. glabrata* and their SFFM on progressive sperm motility is presented in figure 2. Immediately, when incubation began, both strains significantly decreased sperm motility, and increasing the time of incubation increased the negative effect, although this was higher when *C. albicans* strains (81%, p<0.0001) or their SFFM were incubated (58%, p<0.001) with respect to the control.

**Figure 3.**
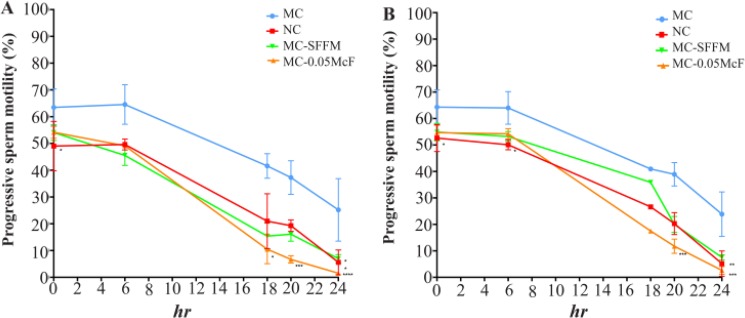
Effect of *Candida* spp. on sperm capacitation process. *C. albicans* (a) and *C. glabrata* (b) on progressive motility during sperm capacitation process. MC: Capacitating medium, MC-FS: Capacitating medium and 100 *μl* SFFM, MC-0.05 McF: Capacitating medium and Candida =0.05 McF, NC: Non-capacitating medium. Data are presented as median and a range of ten (*C. albicans*) or seven (*C. glabrata*) independent experiments with duplicate determinations. *: p<0,05; **: p<0,01; ***: p<0,001, ****: p<0,0001. SFFM: Soluble factors of fungal metabolism

During the capacitation assays, the greatest effect was demonstrated with the strain of *C. albicans* that reduced motility to 94% (MCA-0.05 McF, p<0.0001) while *C. glabrata* reduced it to 89% (MCG-0.05 McF p<0.001) (Figure 3).

The effect of 0.5 McF SFFM of the two yeast strains on the sperm functional parameters did not show statistical differences. However, *C. albicans* decreased early apoptotic cells by 14%, but increased late apoptosis cells by 67%, necrotic cells by 4% and DNA fragmentation index by 4.3%. As for *C. glabrata*, it increased early apoptosis cells by 69% and late apoptosis to 37%, but it decreased necrotic cells to 17% and DNA fragmentation index to 19% (Table 1).

**Table 1.** Effect of the 0.5 McF soluble factors of *Candida* spp. on the functional parameters

**Parameter median (range)**	**Control**	***C. albicans***	***C. glabrata***
**Early apoptosis (%)**	4 (2,2–9,9)	3,42 (1,7–11) ^a^	6,76 (4,6–7,9) ^a^
**Late apoptosis (%)**	7,38 (4,2–25,8)	12,34 (7,6–27,1) ^a^	10,14 (2,2–41,5) ^a^
**Cells with high ΔΨ_m_ (%)**	77,65 (53,1–81,4)	74,2 (48,5–76,1) ^a^	77,35 (49,2–79,9) ^a^
**Lipoperoxidation of membrane (%)**	60,4 (0,38–98,6)	59,9(0,38–86,8) ^a^	56,1 (0,51–73,6) ^a^
**Cells with intact membrane (%)**	73,9 (36,9–84,5)	70,35 (54,8–76,7) ^a^	74,4 (52,9–85,3) ^a^
**Necrotic cells (%)**	21,7 (12,9–23,6)	24,7(21,7–25,7) ^a^	17,9 (12,7–26,4) ^a^
**Reactive oxygen species production (%)**	70,7 (60,1–81,1)	67 (62–80,7) ^a^	73,9 (47,4–78,6) ^a^
**DNA fragmentation index (%)**	30,15 (9,3–52,5)	31,45 (9,3–56,6) ^a^	24,5 (9,5–63,9) ^a^

a: *vs* control p>0.05 (No statistical significant def.) Mitochondrial membrane potential (ΔΨ_m_)

**Figure 4.**
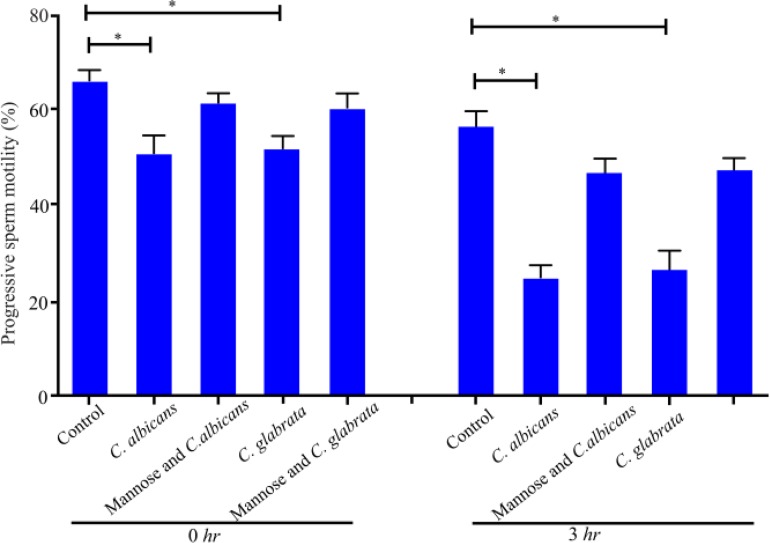
Mannose receptor mediates sperm cell interaction with *Candida* spp. Effect of *C. albicans* and *C. glabrata* on progressive motility after sperm pre-incubation with mannose for 0 or 3 *hr*. Data are presented as median and a range of three independent experiments with duplicate determinations. *: p<0,05

Finally, the effect of *C. albicans* and *C. glabrata* on progressive motility after 1 *hr* of sperm preincubation with mannose, at 0 and 3 *hr* with *Candida* spp. is shown in figure 4. Pre-incubation of sperm with mannose reduced the effect of yeast on mobility and no statistically significant differences were observed with control. Treatment with *C. albicans* or *C. glabrata* and without mannose decreased sperm motility in both cases (0 and 3 *hr*) (p<0.05) compared to the control.

## Discussion

In the present study, the effect of *C. albicans* and *C. glabrata* on sperm quality was evaluated. *Candida* spp. strains and their SFFM negatively affect the motility and sperm viability *in vitro* after 3 and 24 *hr*, and although both yeast strains significantly affect conventional parameters, *C. albicans* has a greater negative effect due to its virulence factors. However, with both strains, the effect is associated with yeast concentration and time of incubation. Three hours are sufficient to significantly modify the percentage of viable and progressive spermatozoa. These effects suggest that the sperm alterations caused by *Candida* spp. may be of greater importance during the fertility consultation.

Men infertility is associated with a decrease in the seminal quality that is frequently affected by uropathogenic or commensal microorganisms that colonize the male and female urogenital tract. Fungal infections are mostly asymptomatic and *Candida* spp. is the most common cause of fungal infections. However, this yeast has a high prevalence in infections of the female reproductive tract which can affect semen quality. Few studies have evaluated the effect of *Candida* spp. on seminal quality (8, 12–14).

The most frequently examined yeast until 2009 due to its prevalence was *C. albicans* (11); among the main virulence factors expressed by this microorganism are the cellular morphology, phenotypic change, extracellular enzymatic activity (proteases and phosphatases) and adhesion factors (11). *In vitro* studies have shown that yeasts and their filtrates or SFFM inhibit sperm motility, and this effect has been associated with the concentration and duration of contact (8, 13). Similarly, in the study published by Tuttle et al., *C. albicans* significantly reduced progressive sperm motility and form to nonspecific aggregates after 2 and 4 *hr*, suggesting that *C. albicans* affects male fertility potential (12).

Likewise, Tian et al. reported that *C. albicans* and its SFFM affect sperm morphology, causing the breakdown of the acrosome, inducing the formation of vacuoles in the head of the spermatozoa, damaging the middle piece and altering the normal function of mitochondria; in addition, these authors reported the formation of nonspecific aggregates (13). Rennemeire et al. reported that SFFM from *C. albicans* induces loss of acrosome and sperm viability (24), and Burrello et al. re ported that *C. albicans* sperm interaction increases the DNA fragmentation index and inhibits the fertilization process during assisted reproduction techniques (14). Burrello et al. later demonstrated alterations in sperm motility, mitochondrial membrane potential and cellular apoptosis after 3 and 24 *hr* of incubation with *C. albicans* (8). In contrast, Berktas et al. evaluated the effect of *C. albicans* on sperm motility at various concentrations, but found no influence of this microorganism on the seminal parameters (25).

With respect to sperm DNA damage, it is important to remark that several studies report that sperm incubation at 37°*C* during 3 *hr* is associated with DNA damage (26, 27). In our case, the treatment during 3 *hr* with the SFFM was not different in relation to the control. However, the higher result of SCSA test could be originated by this incubation.

On the other hand, no reports examined the effect of *C. glabrata* on seminal quality, although *C. glabrata* causes balanitis and orchitis (25). During the last three decades, an increase in the number of cases of vaginal candidiasis, candidemia and systemic fungal infections caused by this species of yeast has occurred. It has even been considered an emerging pathogen, although it appears less virulent than other *Candida* species, due to the absence of pseudohifas, structures that increase the adherence and penetration of the fungus into tissues and cultures. However, it is known that *C. glabrata* produces proteases and presents hydrophobicity on its cell surface, allowing adherence similar to that of *C. albicans* (28).

Regarding the functional parameters examined in the present study, no statistical differences were found. However, similar to what was reported in the literature (8), SFFM of *C. albicans* and *C. glabrata* affects some functional parameters. No reports regarding how the presence of *Candida* spp. affects reactive oxygen species have appeared and in this study, *C. albicans* decreases while *C. glabrata* increases them. In addition, *C. glabrata* decreases the lipoperoxidation of the sperm membrane.

On the other hand, during sperm travel to the female reproductive tract, sperm cells undergo the capacitation process. In the present study, *Candida* spp. strains, *C. albicans* and *C. glabrata* induce changes in the motility of the sperm during the capacitation process. If this same process occurs *in vivo*, the possibility to fertilize the oocyte decreases.

*Candida* spp. has mannan in the form of a glycoprotein that consists mainly of mannose residues and spermatozoa have receptors for this carbohydrate (29–32); these interactions explain these aggregations that are not present in mannose-pre-incubated samples. Therefore, in the future, a new methodology with blocked mannose receptor could be used to decrease the negative effects of *Candida* infection. In addition, there are several limitations in this study that should be noted. Sperm morphology and reversible effects of *Candida* spp. were not determined. Therefore, more studies are needed to determine the effects of *in vitro* or *in vivo Candida* spp. on motility, viability and sperm morphology.

## Conclusion

The presence of *C. albicans, C. glabrata* and their SFFM affect seminal parameters, and this effect is related to incubation time and yeast concentration.

## References

[1] World Health Organization World Health Organization laboratory manual for the examination and processing of human semen. 5th ed. Geneva, Switzerland: World Health Organization 2010 286 p.

[2] Alsterholm M Flytström I Leifsdottir R Faergemann J Bergbrant IM Frequency of bacteria, Candida and malassezia species in balanoposthitis. Acta Derm Venereol. 2008; 88( 4): 331– 6. 18709300 10.2340/00015555-0478

[3] Puerta Suárez J Villegas Castaño A Serna Quintana GJ Martínez A Romero Palacio J Giraldo M [Spermoculture: Bacterial growth in ejaculation and its relationship with the seminal parameters]. Rev Chil Obstet Ginecol. 2015; 80( 1): 33– 40. Spanish.

[4] Cardona Maya WD Du Plessis SS Velilla PA Semen as virus reservoir? J Assist Reprod Genet. 2016; 33( 9): 1255– 6. 27241530 10.1007/s10815-016-0747-8 PMC5010805

[5] Puerta Suárez J Cardona Maya WD [Prevalence of Chlamydia trachomatis, Neisseria gonorrhoeae and Ureaplasma urealyticum in semen samples: effects on sperm quality]. Urología Colomb. 2016; 25( 3): 219– 24. Spanish.

[6] Ahmad G du Plessis SS Agarwal A Sexually Transmitted Infections and Impact on Male Fertility. In: Gunasekaran K Pandiyan N editors. Male Infertility: A Clinical Approach. New Delhi: Springer India; 2017 p. 167– 83.

[7] Brown GD Denning DW Gow NA Levitz SM Netea MG White TC Hidden killers: human fungal infections. Sci Transl Med. 2012; 4( 165): 165rv13. 10.1126/scitranslmed.3004404 23253612

[8] Burrello N Salmeri M Perdichizzi A Bellanca S Pettinato G D'Agata R Candida albicans experimental infection: effects on human sperm motility, mitochondrial membrane potential and apoptosis. Reprod Biomed Online. 2009; 18( 4): 496– 501. 19400990 10.1016/s1472-6483(10)60125-3

[9] Wise GJ Talluri GS Marella VK Fungal infections of the genitourinary system: manifestations, diagnosis, and treatment. Urol Clin North Am. 1999; 26( 4): 701– 18, vii. 10584612 10.1016/s0094-0143(05)70212-3

[10] Aridogan IA Izol V Ilkit M Superficial fungal infections of the male genitalia: a review. Crit Rev Microbiol. 2011; 37( 3): 237– 44. 21668404 10.3109/1040841X.2011.572862

[11] Achkar JM Fries BC Candida infections of the genitourinary tract. Clin Microbiol Rev. 2010; 23( 2): 253– 73. 20375352 10.1128/CMR.00076-09 PMC2863365

[12] Tuttle JP Jr Bannister ER Derrick FC Interference of human spermatozoal motility and spermatozoal agglutination by Candida albicans. J Urol. 1977; 118( 5): 797– 9. 335090 10.1016/s0022-5347(17)58197-5

[13] Tian YH Xiong JW Hu L Huang DH Xiong CL Candida albicans and filtrates interfere with human spermatozoal motility and alter the ultrastructure of spermatozoa: an in vitro study. Int J Androl. 2007; 30( 5): 421– 9. 17298548 10.1111/j.1365-2605.2006.00734.x

[14] Burrello N Calogero AE Perdichizzi A Salmeri M D'Agata R Vicari E Inhibition of oocyte fertilization by assisted reproductive techniques and increased sperm DNA fragmentation in the presence of Candida albicans: a case report. Reprod Biomed Online. 2004; 8( 5): 569– 73. 15151722 10.1016/s1472-6483(10)61104-2

[15] Aitken RJ Sperm function tests and fertility. Int J Androl. 2006; 29( 1): 69– 75. 16466526 10.1111/j.1365-2605.2005.00630.x

[16] Gil-Villa AM Cardona-Maya W Agarwal A Sharma R Cadavid A Role of male factor in early recurrent embryo loss: do antioxidants have any effect? Fertil Steril. 2009; 92( 2): 565– 71. 18829003 10.1016/j.fertnstert.2008.07.1715

[17] Gil-Villa AM Cardona-Maya W Agarwal A Sharma R Cadavid A Assessment of sperm factors possibly involved in early recurrent pregnancy loss. Fertil Steril. 2010; 94( 4): 1465– 72. 19540481 10.1016/j.fertnstert.2009.05.042

[18] Rodríguez E Gil-Villa AM Aguirre-Acevedo DC Cardona-Maya W Cadavid AP [Evaluation of atypical semen parameters in individuals whose couples had a history of early recurrent embryo death: in search for a reference value]. Biomedica. 2011; 31( 1): 100– 7. Spanish. 22159488 10.1590/S0120-41572011000100012

[19] Mayorga-Torres BJ Cardona-Maya W Cadavid Á Camargo M [Evaluation of sperm functional parameters in normozoospermic infertile individuals]. Actas Urol Esp. 2013; 37( 4): 221– 7. Spanish. 23246107 10.1016/j.acuro.2012.06.008

[20] Mayorga-Torres BJ Camargo M Agarwal A du Plessis SS Cadavid ÁP Cardona Maya WD Influence of ejaculation frequency on seminal parameters. Reprod Biol Endocrinol. 2015; 13: 47. 25994017 10.1186/s12958-015-0045-9 PMC4445565

[21] Lalinde-Acevedo PC Mayorga-Torres BJM Agarwal A du Plessis SS Ahmad G Cadavid ÁP Physically Active Men Show Better Semen Parameters than Their Sedentary Counterparts. Int J Fertil Steril. 2017; 11( 3): 156– 65. 28868837 10.22074/ijfs.2017.4881 PMC5582143

[22] Mayorga-Torres BJM Camargo M Cadavid ÁP du Plessis SS Cardona Maya WD Are oxidative stress markers associated with unexplained male infertility? Andrologia. 2017; 49( 5) 10.1111/and.12659 27506165

[23] Glander HJ Schaller J Binding of annexin V to plasma membranes of human spermatozoa: a rapid assay for detection of membrane changes after cryostorage. Mol Hum Reprod. 1999; 5( 2): 109– 15. 10065865 10.1093/molehr/5.2.109

[24] Rennemeier C Frambach T Hennicke F Dietl J Staib P Microbial quorum-sensing molecules induce acrosome loss and cell death in human spermatozoa. Infect Immun. 2009; 77( 11): 4990– 7. 19687207 10.1128/IAI.00586-09 PMC2772518

[25] Berktas M Aydin S Yilmaz Y Cecen K Bozkurt H Sperm motility changes after coincubation with various uropathogenic microorganisms: an in vitro experimental study. Int Urol Nephrol. 2008; 40( 2): 383– 9. 17934792 10.1007/s11255-007-9289-4

[26] Matsuura R Takeuchi T Yoshida A Preparation and incubation conditions affect the DNA integrity of ejaculated human spermatozoa. Asian J Androl. 2010; 12( 5): 753– 9. 20562894 10.1038/aja.2010.46 PMC3739315

[27] Nabi A Khalili MA Halvaei I Roodbari F Prolonged incubation of processed human spermatozoa will increase DNA fragmentation. Andrologia. 2014; 46( 4): 374– 9. 24689689 10.1111/and.12088

[28] del Valle GM Candida glabrata: un patógeno emergente. Biociencias. 2016; 10( 1): 89– 102.

[29] Cardona-Maya WD Cadavid AP [Evaluation of the role of the monosaccharides, mannose and N-acetylglucosamine in the induction of the acrosome reaction in human spermatozoa]. Actas Urol Esp. 2005; 29( 7): 676– 84. Spanish. 16180318 10.1016/s0210-4806(05)73318-0

[30] Cardona-Maya W Velilla P Montoya CJ Cadavid A Rugeles MT Presence of HIV-1 DNA in spermatozoa from HIV-positive patients: changes in the semen parameters. Curr HIV Res. 2009; 7( 4): 418– 24. 19601777 10.2174/157016209788680570

[31] Cardona-Maya W Velilla PA Montoya CJ Cadavid Á Rugeles MT In vitro human immunodeficiency virus and sperm cell interaction mediated by the mannose receptor. J Reprod Immunol. 2011; 92( 1–2): 1– 7. 22015004 10.1016/j.jri.2011.09.002

[32] Cardona-Maya W López-Herrera A Velilla-Hernández P Rugeles MT Cadavid AP The role of mannose receptor on HIV-1 entry into human spermatozoa. Am J Reprod Immunol. 2006; 55( 4): 241– 5. 16533334 10.1111/j.1600-0897.2005.00340.x

